# Rationale for using insensitive quality control rules for today's hematology analyzers

**DOI:** 10.1111/j.1751-553X.2010.01229.x

**Published:** 2010-12

**Authors:** G S Cembrowski, B Smith, D Tung

**Affiliations:** Department of Laboratory Medicine and Pathology, University of Alberta HospitalEdmonton, Alberta, Canada

**Keywords:** Quality control, hematology, imprecision

## Abstract

Diverse approaches have been used to assure the analytical quality of automated hematology; as such, there is great variation in their error detection capabilities. We summarize the intralaboratory performance of a cohort of Sysmex XE-2100’s running e-Check hematology quality control (QC). The imprecisions of a median performing (50th percentile imprecision) and more imprecise [15th percentile (15P) imprecision] Sysmex XE-2100 are compared with measures of total allowable error (regulatory and physiologically based) to obtain multiples of the usual imprecision that must be detected to prevent the hematology analyzer from producing medically unacceptable results. The resultant large multiples of the usual imprecision (*s*) demonstrate the need for insensitive QC rules employing very broad control ranges, control rules that have been implicitly supported by hematology analyzer manufacturers for the last several decades. For today’s highly precise hematology analyzers, the following control rules are strongly advised: 1_3.5*s*_, 1_4*s*_ and 1_4.5*s*_ rules (violated if a single control observation exceeds either its ±3.5, ±4.0 and ±4.5*s* limits, respectively). In order for the hematology laboratory to totally embrace expanded QC limits, manufacturers must make available their instruments’ usual and poorer (e.g. the 15P performance) imprecision’s. Users of hematology analyzers that require more sensitive but less specific rules to prevent the reporting of clinically erroneous data are advised to acquire more precise (and thus more dependable) instrumentation.

## Introduction

Diverse approaches have been used to assure the analytical quality of automated hematology analyzers. These approaches encompass the re-analysis of retained patient specimens (Cembrowski *et al.*, 1988; Hackney & Cembrowski, 1990) averaging selected consecutive patient measurements including red blood cell indices (patient moving averages) (Bull *et al.*, 1974; Koepke & Protextor, 1981; Levy, Hay & Bull, 1986); as well as the use of stabilized hematology control products. Each of these procedures has certain advantages and disadvantages in terms of practicality and error detection capabilities. While the re-analysis of retained specimens is inexpensive, this practice is generally used to detect short-term (within day) trends and shifts. Averages of patient data are susceptible to changes in the population of patients being analyzed (Cembrowski & Westgard, 1985); the ambient room temperature and even the temperature of the hematology reagents (Cembrowski, Hodgson & Etches, 2002). Commercial quality control (QC) materials are often regarded as expensive, variably sensitive in the detection of analytical errors and subject to artifactual error because of constituent instability.

The error detection capabilities of these procedures differ greatly and are best described by power function curves, which are plots of error detection *vs.* the size of error. Westgard has provided power function curves for control rules based on the analysis of reference sample materials (commercial QC material) (Westgard & Groth, 1979). Cembrowski and others have developed power function plots for QC procedures using retained patient specimens and moving averages of patient data (Cembrowski *et al.*, 1988; Cembrowski & Westgard, 1985; Lunetzky & Cembrowski, 1987). These power function curves show that reference sample QC procedures will detect analytical error more quickly and more accurately than either retained patient samples or moving averages (Westgard & Cembrowski, 1990).

While virtually all laboratories in developed countries analyze commercial control materials for hematology QC, there is significant variation in the setup and interpretation of the hematology QC. The control limits are variable with some laboratories using the limits supplied by the manufacturer, and others using control limits that are derived statistically from preliminary analysis of the new QC material. The frequency of control material analysis varies. Some laboratories analyze controls every 8 or 12 h. Other laboratories ‘bracket’ groups of patient specimens with QC specimens or retained, previously analyzed specimens. There is also significant variation in the control rules and control procedures (combinations of QC rules applied in a defined manner) used to interpret the QC data. Table 1 shows some popular QC rules.

**Table 1:** Examples of quality control rules (*s*, standard deviation)

Rule	Definition	Comments
1_2*s*_	Use as a rejection or warning when one control observation exceeds the *x* ± 2*s* control limits; usually used as a warning	Overused. Should only be used with manual assays with low number of analytes/control materials
1_3*s*_	Reject a run when one control observation exceeds the *x* ± 3*s* control limits	Detects increased imprecision (random error) and shifts (systematic error)
1_3.5*s*_	Reject a run when one control observation exceeds the *x* ± 3.5*s* control limits	Detects increased imprecision (random error) and shifts (systematic error)
1_4*s*_	Reject a run when one control observation exceeds the *x* ± 4*s* control limits	
1_4.5*s*_	Reject a run when one control observation exceeds the *x* ± 4.5*s* control limits	
2_2*s*_	Reject a run when two consecutive control observations are on the same side of the mean and exceed the *x*+2*s* or *x*− 2*s* control limits	Detects shifts (systematic error), may be applied across analytic runs (within control materials) and within analytic runs (across control materials)
2/3_2*s*_	Reject a run when two of three control observations are on the same side of the mean and exceed the *x* + 2*s* or *x* − 2*s* control limits	

A 1994 College of American Pathologists Q-Probe surveyed the QC practices in 505 primarily US health care institutions (39% teaching and 61% nonteaching) (Howanitz, Tetrault & Steindel, 1997). Overall, for every 100 specimens analyzed, approximately seven were QC specimens. 0.36% of runs were rejected and resulted in 0.3% of patient specimens being rerun and 25-minute investigational delays for each rejected run. For every 100 specimens measured, only 91 analyses were billable. On average, 40% of participants applied more than one QC rule; all told, 15 different QC rules were used to detect analytical errors in hemoglobin (Hgb). While participants tended to use QC rules that detected shifts or biases, these types of errors were in the minority and caused QC exceptions only 16% of the time. The survey indicated a lack of adherence to QC policy. While 31% of the laboratories had a policy to repeat all rejected patient samples, only 4% adhered to this policy. Sadly, 51% of participants excluded rejected control values from statistical analysis and 66% of participants re-measured the same control sample when an exception occurred rather than measuring a new specimen. The study authors concluded that ‘laboratorians have difficulty in following QC rules because they are complex, tedious to follow, and in some cases impractical’.

It is incongruous that the quality of today’s multichannel hematology analyzers is generally undisputed, yet the practice of hematology QC is heterogeneous and seemingly arbitrary. It may be that these analyzers are robust and usually produce clinically acceptable data and that many of today’s QC procedures are nonspecific and lead to nonproductive practices. In this study, we evaluate the stability of e-Check hematology QC product as analyzed by a cohort of laboratories using a representative robust hematology analyzer, the Sysmex XE-2100 (Johnson *et al.*, 2002). We summarize the intralaboratory performance of this cohort of Sysmex XE-2100’s. The imprecision of a median performing analyzer (50th percentile imprecision) and a less than average analyzer [15th percentile (15P) imprecision] are compared with estimates of maximum allowable error (MAE). The MAE corresponds to the largest amount of error that can be added to a test result before the test result becomes unfit for medical use. While the MAE should be considered a total error and includes pre-analytic and analytic error, laboratorians usually focus on analytic error. Many American laboratorians interchange MAE and the US Clinical Laboratory Improvement Amendments (CLIA; Medicare Medicaid and CLIA programs, 1992) limits for proficiency testing. As the CLIA error limits are extremely broad, somewhat subjective, and not defined for many analytes, physiologically based estimates of MAE (Ricos *et al.*, 1999) have been embraced by the laboratory community.

The ratio of MAE to the analytical imprecision (CV_a_) (MAE:CV_a_) dictates the QC rule that should be used to detect analytically important error. A high ratio (exceeding 4–5) indicates a very tightly controlled analytical process. For such high ratios, QC rules such as the 1_3.5*s*_, 1_4*s*_ and 1_4.5*s*_ are adequate. For MAE:CV_a_ values of 3–4, tighter control limits are required to detect analytically significant error. A control procedure combining the 1_3*s*_ and the 2_2*s*_ control rules will generally suffice for these intermediate ratio tests.

## Materials and methods

The Sysmex XE-2100 is an automated discrete hematology analyzer designed for high-volume clinical laboratory testing (maximum throughput of 150 samples/h). It provides a 14-parameter hemogram, a 5-part leukocyte differential, reticulocyte analysis including immature reticulocyte fraction, and a nucleated red blood cell (NRBC) count. Differential parameters, reticulocyte analysis, and NRBC counts are determined using flow cytometry, a semi-conductor laser and fluorescent dyes (Walters & Garrity, 2003). The Sysmex XE-2100 also measures and charts 16 other detector control parameters. Samples may either be run in the automated aspiration (closed) sampling mode using a sample volume of 200 μl or in the manual (open) sampling mode using a 130 μl sample volume.

The Sysmex XE-2100 uses a three level control product, Sysmex e-Check, for process QC. This material consists of a stabilized whole blood mixture of human erythrocytes, human and simulated leukocytes and platelets (PLT). If promptly refrigerated after each use, the material has a 73-day closed vial stability and a 7-day open vial stability. Table 2 shows the target means for the three levels of control material. The QC materials’ basophil counts are so elevated that they are not incorporated into the total white blood cell (WBC) count.

**Table 2:** Package Insert Targets for Sysmex e-Check lot no. 2197

	Mean concentrations		
	
	Level 1	Level 2	Level 3
Baso#, 10^9^/l	1.4	4.2	12.2
Baso%	58.7	62.8	69.5
Eo#, 10^9^/l	0.2	0.6	1.8
Eo%	7.6	9.2	10.2
Hct, %	17.6	36.7	47.0
Hgb, g/dl	6.0	12.8	16.8
IRF, %	28.8	27.6	25.1
Lymph#, 10^9^/l	1.1	2.5	5.3
Lymph%	44.2	36.9	30.4
MCH, pg	25.8	29.3	32.7
MCHC, g/dl	34.0	35.0	35.7
MCV, fl	75.9	83.9	91.4
Mono#, 10^9^/l	0.3	0.7	2.2
Mono%	14.0	11.1	12.3
MPV, fl	9.5	9.7	9.8
Neut#, 10^9^/l	0.8	2.8	8.3
Neut%	34.3	42.7	47.2
PLT, 10^9^/l	53.0	206.0	464.0
PLT-O, 10^9^/l	61.0	201.0	458.0
RBC, 10^12^/l	2.33	4.37	5.14
RDW-SD, fl	42.4	41.8	46.7
Ret#, 10^12^/l	0.2	0.1	0.1
Ret%	7.4	3.3	1.1
WBC, 10^9^/l	2.4	6.6	17.5

% indicates the percentage of WBC (white blood cells) represented by the white cell moiety in question, e.g. Baso% is the percentage of WBC represented by basophils; #, the absolute count of the white cell moiety, e.g. Baso# represents the absolute number of basophils/l; PLT-O, optical platelet count; RBC, red blood cell; IRF, immature reticulocyte fraction.

The QC data are transmitted regularly from the Sysmex XE-2100 to the Sysmex Insight Program. This program provides calculations of the individual laboratory’s mean and SD for each control and analyte as well as corresponding interlaboratory statistics. These calculations are continuously available via the World Wide Web. Group summary reports are also produced at the end of each 30-day period. We obtained two 30-day summaries of submitted data for Sysmex e-Check lot number 2197 from 121 different laboratories in pdf format (Adobe Software, San Jose, CA, USA) for the periods of July 17, 2002 to August 15, 2002 (period 1) and August 16, 2002 to September 20, 2002 (period 2). These summaries were converted into text files and the text data extracted into Microsoft Access, Crystal Reports and Microsoft Excel to provide data summaries and graphs.

We tabulated the individual Sysmex Insight intralaboratory CV for each analyte and determined the following CV: 10P, 15P, 50P, 85P and the 90P, 95P and the 99P with the highest percentiles corresponding to the most precise performance. Graphs were constructed showing the 10–99P CV for the analytes shown in Table 2. Periods 1 and 2 were plotted separately to more easily evaluate control product stability.

**Figure 5:**
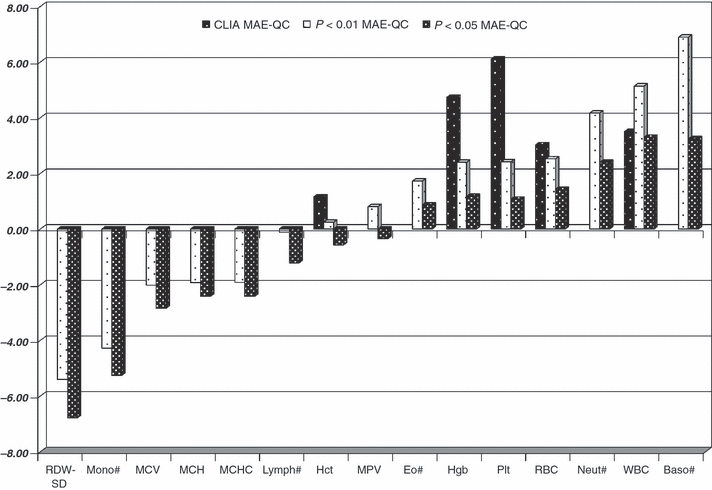
Difference between the maximum allowable error and quality control limits for 50th percentile analyzers (level 2 control product).

Two types of MAE estimates were used. In addition to the U.S. CLIA proficiency testing limits (Medicare Medicaid and CLIA programs, 1992), we also used two set of allowable errors based on physiological variation (Ricos *et al.*, 1999). One set encompassed 95% of observations and the other, 99%. We calculated the MAE:CV_a_ ratios for both the 15P and 50P CV. We also calculated the error magnitude (measured in number of SD's) required in the results before an error would exceed the manufacturer’s limits. Finally, the differences between the MAE:CV_a_ ratios and the QC error magnitudes were calculated (Figure 5).

## Results

The participants consisted of hospital and nonhospital laboratories. Most laboratories ran all three levels of control material. In period 1, reports were available for 115 analyzers from 88 laboratories, 24 of which used multiple analyzers. Six analyzers ran the QC both in the ‘closed’ and ‘open’ sampling mode, for a total of 121 sets of data. About 110 sets of data were obtained in the ‘closed’ mode and the remainder was run ‘open’. Three laboratories (six analyzers) did not provide period 2 control data. On average, 60 controls were analyzed at each control level.

In period 2, reports were available for 153 analyzers from 118 laboratories; 32 of which used multiple analyzers. Nine analyzers ran QC both in the ‘closed’ and ‘open’ sampling mode, for a total of 162 sets of data. One hundred and forty nine sets of data were obtained in the ‘closed’ mode and the remainder was run ‘open’.

On average, 70 controls were analyzed at each control level. In a sample of 50 hospital laboratories that maintained 24-h services, 46% ran QC three times per day, 34% ran two times per day, 14% four times a day and 6% ran QC only once a day.

**Figure 1:**
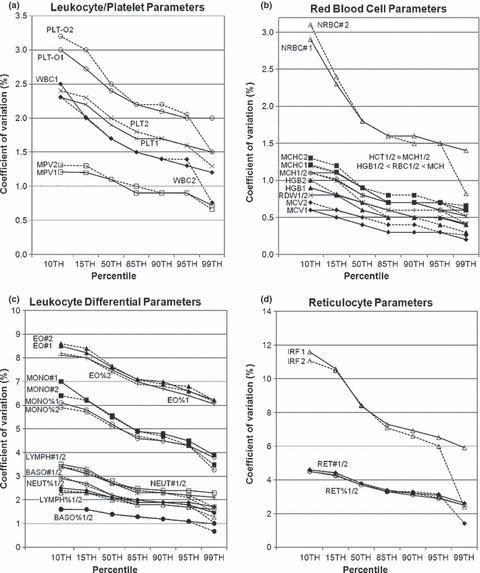
(a–d) Comparison of period 1 and 2 CV for 10, 15, 50, 85, 90, 95 and 99 percentile Sysmex XE-2100 instruments for leukocytes/platelets (a), red blood cell parameters (b), leukocyte differential parameters (c) and reticulocyte parameters (d).

**Figure 2:**
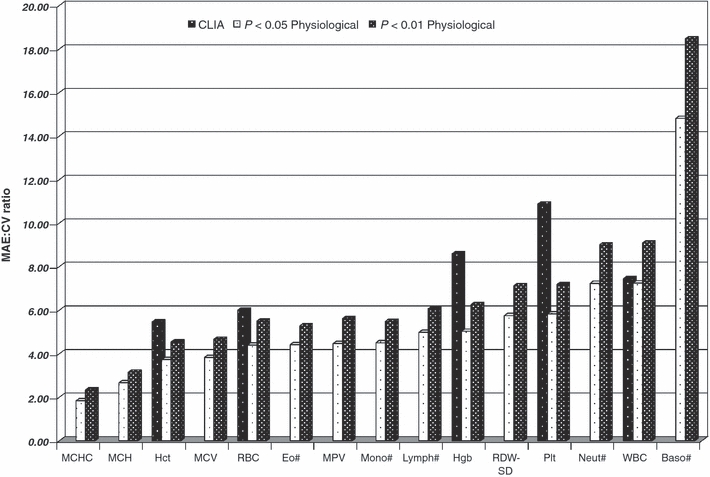
Maximum allowable error:CV_a_ ratios for 15th percentile CV analyzers. [Correction added on 22 September 2010, after first online publication: The wrong data for Figure 2 was used during typesetting and this has been corrected.]

**Figure 3:**
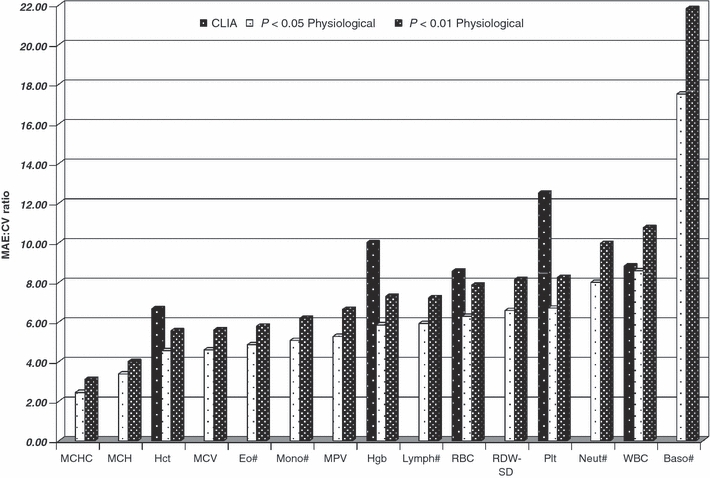
Maximum allowable error:CV_a_ ratios for 50th percentile CV analyzers.

**Figure 4:**
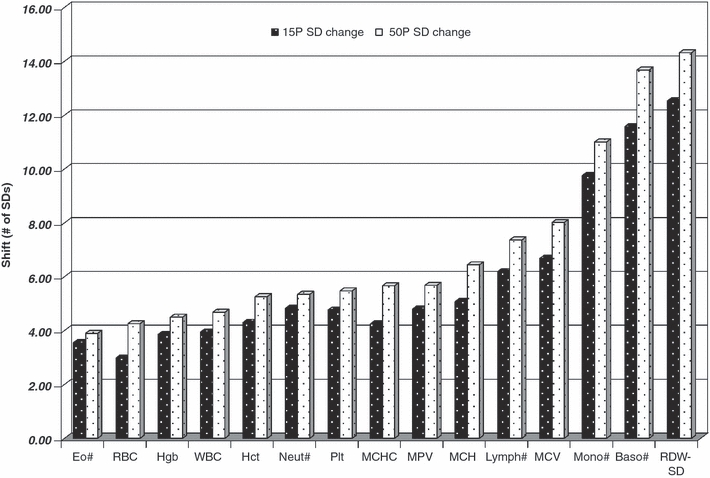
Shift required (in number of SD) before error is signaled by quality control (QC) result being out of manufacturer QC limits (level 2 control product).

Figure 1a–d shows the comparison between period 1 and period 2 imprecisions for leukocytes/PLT, red blood cell parameters, leukocyte differential parameters and reticulocyte parameters, respectively. The period 1 and period 2 imprecisions were not statistically different for all tests except for MCV (*P*< 0.01). Figures 2 and 3 show that the majority of parameters have an MAE:CV_a_ ratio >4 except for mean corpuscular hemoglobin (MCH), mean corpuscular hemoglobin concentration (MCHC) and mean corpuscular volume (MCV). Figure 4 displays the shift from the mean (measured in number of SD's) required in QC samples for the manufacturer QC limits to be violated (15P and 50P; level 2). For example, a Hgb result for a level 2 QC sample run on a 50P analyzer that is 4 SD outside the expected mean will result in an error.

Figure 5 shows the difference between the MAE and the manufacturer’s QC limits (expressed in multiples of SD) for the 15P analyzers running level 2 QC samples. Negative differences indicate that the QC limits are wider than the MAE limits and that any out of QC limits value will also violate the MAE limits. More importantly, in these cases some errors may exceed MAE limits without causing QC violations, leading to unsuspected reporting of erroneous results. The positive differences indicate that QC limits are narrower than MAE limits. In this scenario, it is not possible to exceed the MAE limits without also violating the QC limits, therefore all significant errors should be detectable and potentially correctable.

## Discussion

### Imprecision

The period 1 and period 2 CV are somewhat larger than those demonstrated in an early XE-2100 evaluation (Walters & Garrity, 2003). Published evaluations of multichannel hematology instruments are generally performed by few individuals on a single analyzer over a 20-day period using a limited number of samples. Our work represents multiple analyzers run over 60-day periods and reflects variation in multiple instruments, reagent lot numbers and operators. Figure 1 shows that the period 1 and 2 CV’s are almost superimposable. While the CV of MCV increased by 20% in period 2, the resultant CV is only 0.5% (level 2–50P) reflecting a very small analytical and even a smaller clinical change. The Sysmex XE-2100 directly measures hematocrit (Hct), RBC and Hgb, while MCH, MCHC and MCV are calculated using the aforementioned measured values. Therefore the imprecisions of MCH, MCHC and MCV reflects the collective imprecisions of the Hct, RBC and Hgb measurements.

### Ratio of allowable error to imprecision and quality control limits

When using the CLIA limits, the MAE:CV_a_ ratios exceed 5 for all analytes for the 15P (below average) analyzers (Figure 2). When the samples are run on a 50P (average) analyzer, the ratios exceed 6 (Figure 3). When using the physiologic variation limits, the MAE:CV_a_ ratios of most of the analytes exceed 4 for 15P and 50P instruments. While the MCV and Hct ratios cluster around 4, the MCH and MCHC ratios are closer to 2. Most of the QC limits used by the manufacturer appear to approximate the 3.5, 4 and 4.5*s* control limits (Figure 4). However, for absolute lymphocyte counts, MCH and MCV the control limits range from 4.5 to 8*s*. The control limits are in excess of 10*s* for red cell distribution width (RDW) and absolute basophil/monocyte counts.

### Maximum allowable error quality control limits

Using the CLIA limits, the manufacturer QC limits are narrower than the MAE for the 15P analyzer (Figure 5) as well as the 50P analyzer. Therefore, the relatively insensitive manufacturer QC limits can detect errors in hematocrit, Hgb, PLT, RBC and WBC before a CLIA limit is violated. The QC limits can detect errors before the *P*< 0.05 physiologic limit is exceeded for leukocyte, neutrophil, eosinophil and basophil counts, Hgb, PLT and RBC. With the use of *P*< 0.01 physiologic limits, this list of analytes would also include mean platelet volume (MPV) and Hct. Of these tests, Hgb, PLT, leukocyte count and neutrophil number, are probably the most clinically useful and most commonly ordered tests. With reference to those tests for which both physiologic limits are narrower than the QC limits, three of the tests, MCV, MCH and MCHC are calculated by the Sysmex. We have found that the investigation of deviations in calculated parameters is not productive as these deviations usually arise from simultaneous errors in two or more of the constituent measurements.

The QC limits for Hct and MPV appear to approximate the physiologic limits. Unlike Hgb, clinicians generally do not follow Hct serially; however, values outside the reference range elicit attention. Generally, values <0.3 are considered to be clinically significant, which is well below the lower limit of the normal reference range (0.36 and 0.41 for women and men, respectively). Therefore, deviations well in excess of the QC limits ( ± 0.014) are required before clinical intervention is required. For MPV, an uncommonly ordered and rarely reported parameter, the clinically significant changes appear to be at least twice as much as the physiologic limits (Khandekar *et al.*, 2006). Therefore, the QC limits for MPV still have the potential to detect errors before they become clinically relevant. RDW-SD is another parameter that typically prompts further investigation only if it is outside the normal reference range (37–50 fl). Of note, RDW-SD is rarely studied in isolation, but rather in combination with other CBC parameters and cell morphology. RDW-SD also has only limited clinical utility; it is generally increased in iron deficiency anemia and some hemoglobinopathies and usually normal in thalassemia. For these diseases other CBC parameters serve as more reliable markers. Therefore, the broad QC limits for RDW-SD ( ± 3.68 fl) are unlikely to lead to any clinically significant errors. The QC limit for lymphocyte number ( ± 0.3 × 10^9^) is similar to the physiologic limit while the QC limits for monocyte number ( ± 0.4 × 10^9^) are wider. The tight lymphocyte QC limits are adequate for lymphocytosis, where lymphocyte numbers are generally increased well in excess of the QC limits. Unfortunately, the QC limits may be too wide for lymphopenia, monocytopenia and monocytosis. For example, a monocyte count >1 × 10^9^/l is considered clinically significant, therefore, the possibility exists that a negative shift in the results will provide a false negative without violating the QC limits. Monocytes may be elevated in chronic infections and myeloproliferative/myelodysplastic disorders. However, we have other markers such as neutrophil and leukocyte number for infectious processes. Patients with myeloproliferative disorders do not typically present with isolated monocytosis but also demonstrate other cytosis, distinctive cytologic and histologic features, phenotypic changes on flow cytometry and abnormal clinical findings. Therefore, the possibility of clinical significant error appears to be remote.

It is our belief, based on the information in Figures 2, 3 and 5 that the QC limits recommended by the manufacturer are acceptable and can be used with laboratories operating XE-2100s. This recommendation stands for both the below-average and average analyzers. The results of this work will be embraced by most hematologists as they are usually less versed in statistical QC than their medical biochemistry colleagues. The more demanding hematologist, can still use traditional statistical limits. Based on Figures 2 and 3, the 1_4.5*s*_ control rule would be adequate for most analytes and the 1_3.5*s*_ control rule should suffice for Hct.

It is probable that other hematology analyzers are capable of such precise analytic performance. In order for the hematology laboratory to totally embrace expanded QC limits, manufacturers must make available their instruments’ usual imprecision’s as well as well as their instruments’ poor imprecision’s (e.g. the 15P performance). Selection of control rules based on average performance can be misleading; after all, half the analyzers will deliver less than average performance. Use of these expanded rules should markedly reduce the needless re-analysis based on 1_3*s*_ and 2_2*s*_ rule rejections and make the investigation of outlyling QC observations far more rewarding. Users of hematology analyzers who require the use of 1_3*s*_ and 2_2*s*_ to prevent the reporting of clinically erroneous data are advised to replace their analyzers with more precise (and thus more dependable) instrumentation.
